# Application of deep learning based on convolutional neural network model in multimodal ultrasound diagnosis of unexplained cervical lymph node enlargement

**DOI:** 10.3389/fonc.2025.1542265

**Published:** 2025-06-06

**Authors:** Shanshan Jiang, Naiqian Zhang, Chen Li, Lingxia Tong, Xiuhua Yang

**Affiliations:** ^1^ Department of Qunli Ultrasound, The First Affiliated Hospital of Harbin Medical University, Harbin, Heilongjiang, China; ^2^ Department of Ultrasound, Jilin Cancer Hospital, Changchun, Jilin, China

**Keywords:** deep learning, color Doppler flow imaging(CDFI), elastography, lymph node classification, webserver

## Abstract

This study retrospectively analyzed the multimodal ultrasound features and clinical characteristics of 586 patients with unexplained cervical lymphadenopathy who were treated at three hospitals between October 2019 and December 2022. Statistically significant differences were found in the clinical and ultrasound features of all patients, including location, shape, margin, and color Doppler flow imaging (CDFI) (p<0.05). Deep learning models, particularly convolutional neural networks (CNNs), demonstrated great potential in classifying cervical lymph node pathologies using multimodal ultrasound images, including 2D imaging, color Doppler flow imaging (CDFI), and elastography. First, we pre-trained four convolutional neural networks using a public medical image dataset. Then, we fine-tuned the models for three-class classification of lymph nodes into metastatic, lymphoma, and benign using 2D, CDFI, and elastography images from the patients’ lymph nodes. The pre-trained ResNet model performed excellently, with an elastography AUC of 0.925, outperforming other models. Elastography became the most reliable feature extraction dataset, significantly enhancing the model’s accuracy in distinguishing between benign, lymphoma, and metastatic lymph nodes. Ablation experiments showed that pre-training significantly improved accuracy compared to non-pre-trained models. Grad-CAM visualization provided valuable interpretability, revealing how the model focuses on specific areas corresponding to each pathology. Based on this model, we developed a user-friendly server, CV4LymphNode (https://hwwlab.com/webserver/cv4lymphnode). This study highlights the potential of deep learning in accurately classifying cervical lymph node pathologies.

## 1 Introduction

The cervical lymph nodes primarily function to collect lymphatic fluid from the head, thoracic duct, and associated lymphatic vessels. Enlargement of these lymph nodes may indicate the presence of local or systemic diseases. Common conditions associated with cervical lymphadenopathy include reactive hyperplasia, tuberculosis, metastatic malignancies, and lymphoma. Patients frequently seek medical attention due to cervical lymph node enlargement. With the rising incidence and mortality rates of malignancies, accurately assessing the nature of lymph nodes has become critically important for tumor staging, treatment planning, and prognostication (1).

Ultrasound imaging, owing to its high resolution, convenience, and noninvasiveness, serves as the first-line modality for evaluating cervical lymph node disorders. High-frequency ultrasound and color Doppler ultrasound are routinely utilized (2, 3). However, on conventional two-dimensional gray-scale and color Doppler images, certain benign and malignant superficial lymph nodes may exhibit similar features, complicating the distinction between benign and malignant nodes. Consequently, the specificity of differential diagnosis is reduced, making accurate diagnosis more challenging. To address these limitations, strain elastography (SE) has emerged as a potential adjunct. SE has been successfully employed in the evaluation of conditions such as chronic hepatitis and thyroiditis, demonstrating a positive correlation between SE parameters and tissue stiffness (4, 5). Nevertheless, studies investigating the application of ultrasound elastography in assessing abnormal cervical lymph nodes have yielded inconsistent results (6–8). Moreover, reliance on fine-needle aspiration biopsy to determine the pathology of enlarged lymph nodes carries the risk of procedural complications. Thus, there is an urgent need for a noninvasive and accurate method to diagnose unexplained cervical lymphadenopathy (9).

In recent years, the rapid advancement of computer technology has led to significant progress in the application of deep learning in medical imaging. For example, in the classification of ultrasound images, DeepThy-Net constructed a multimodal thyroid cancer classification model using ultrasound and pathological data, achieving an area under the curve (AUC) of 0.905 (10). Another study employed a CNN-long short-term memory (LSTM) network combining elastography, B-mode, and Doppler images, attaining a classification accuracy of 98.26% for pancreatic lesions (11). Training classification models on large datasets of labeled ultrasound images is expected to enhance both diagnostic efficiency and accuracy. For instance, in a study identifying thyroid nodules, radiologists supplemented with ThyGPT significantly outperformed peers using conventional methods in diagnostic sensitivity (12). Similarly, the Y-Net model was shown to assist sonographers in improving the accuracy of classifying metastatic cervical lymph nodes (13).

However, studies applying deep learning specifically to classify lymph node ultrasound images remain scarce, with most research focusing instead on segmentation tasks. Among the few classification studies, one utilized ResNet to classify 1,000 lymph node ultrasound images from 728 patients, achieving an AUC of 0.902 (14). Another study applied Swin Transformer to classify 2,268 images from 1,146 patients into six categories, achieving an accuracy of 80.65% (15). CLA-HDM achieved an AUC of 0.873 in classifying 763 lymph node ultrasound images and improved the diagnostic accuracy of six radiologists with varying levels of experience (16). Nevertheless, these studies often relied on single ultrasound modalities, and their datasets and source codes were not publicly available, limiting their clinical applicability. In addition, recent findings suggest that pretraining on ultrasound images can significantly enhance downstream classification performance (13). Given the availability of several public ultrasound datasets from other anatomical sites, leveraging such datasets for pretraining may offer a promising approach.

In this study, we retrospectively analyzed the multimodal ultrasound imaging characteristics and clinical features of patients with unexplained cervical lymphadenopathy. We developed a lymph node ultrasound image classification model using a pretraining strategy and established a user-friendly web-based platform to provide intuitive and quantitative predictions of lymph node pathology. Our work aims to offer valuable support for rapid clinical diagnosis and treatment decision-making.

## 2 Materials and methods

**Figure 1:**
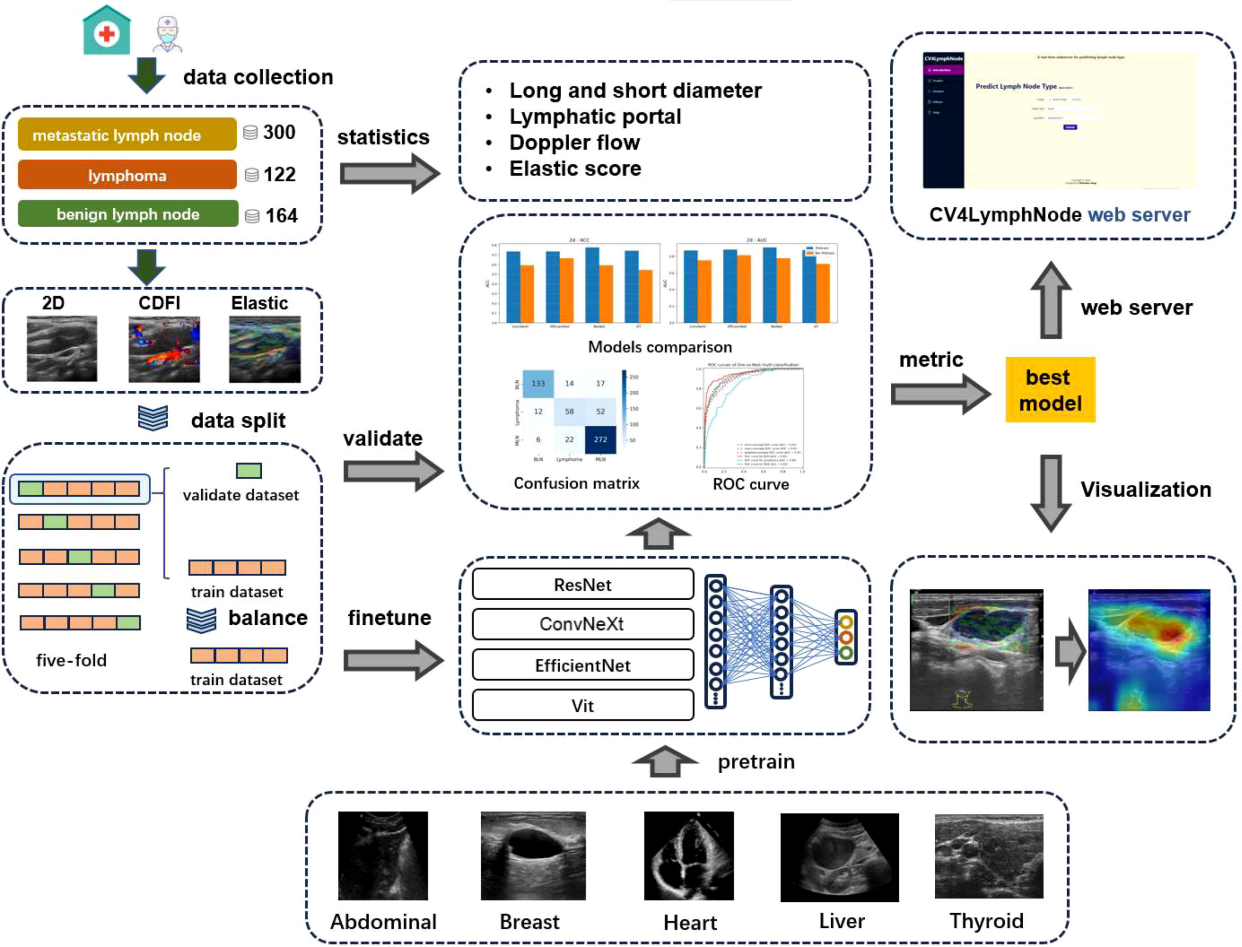
Workflow of the study.

Our workflow is illustrated in **Figure 1**, which proceeds from left to right through statistical analysis of case samples, processing of ultrasound case images, processing of the pretraining dataset, deep learning model architecture and evaluation, interpretable analysis, and the prediction web server.

### 2.1 Patient cases

The study included 586 patients with unexplained cervical lymph node enlargement who received treatment at three hospitals—Jilin Province Cancer Hospital, the Second People’s Hospital of Jilin Province, and the First Affiliated Hospital of Harbin Medical University—from October 2019 to December 2022. All patients provided signed informed consent for ultrasound examination and puncture biopsy, and the study was approved by the ethics committee of each hospital.

Inclusion criteria:

1. The main symptom was cervical lymph node enlargement of unknown cause.
2. All patients underwent puncture or surgical treatment to obtain pathological tissue, and pathological diagnosis results were used as the gold standard.

Exclusion criteria:

1. Presence of metastases in other parts of the body.
2. Lack of pathological examination.
3. History of prior treatment.

Ultrasound images were acquired by two certified physicians, each with more than 10 years of experience in diagnosing lymph node diseases, using GE Doppler ultrasound machines: GE LOGIQ S8 Doppler ultrasound machine (General Electric Company, Boston, USA). A 5–13 MHz linear array probe was used on patients in the supine position, and the ultrasound images were adjusted to achieve the best lymph node imaging effect. Gray scale, color Doppler, and strain elastography static images with typical ultrasonic characteristics on the maximum longitudinal section were obtained for all patients with cervical lymph node swelling. The entire lymph node was dynamically scanned, and the morphological findings were recorded. Lymph nodes are small, oval or bean-shaped organs with a smooth surface. They vary in size from a few millimeters to several centimeters and are typically gray-white or pale pink in color. All ultrasound images were stored for further analysis. To ensure consistency in image analysis, the cervical lymph node images were retrospectively analyzed independently by two doctors in a blind manner, and any differences were discussed to reach a consensus. Finally, the corresponding two-dimensional (conventional ultrasound or grayscale ultrasound) ultrasound, color Doppler, and elastography images were collected for analysis in this study.

According to the Chinese Guidelines for Superficial Organ Ultrasound, gray scale ultrasound was used to evaluate the shape, edge, boundary, and internal echo of the lymph nodes. Color Doppler flow imaging (CDFI) was used to display the characteristics of the internal blood flow of the lymph nodes (17). Ultrasound elastography was employed to determine the hardness of the lesion and surrounding tissue. Based on the standard Asteria method (18), strain elastic imaging (EI) results were scored from 1 to 4. The ultrasonic diagnosis of lymph nodes was determined by the findings from these three modes (19). Subsequently, deep neural networks were used for training and prediction. Conventional ultrasound provides 2D images in black and white that are used to show the structure of the body’s internal organs and tissues. Doppler ultrasound can show the direction and speed of blood flow and is usually color-coded. Elastic ultrasound assesses the elasticity and hardness of a tissue by measuring how much the tissue deforms when pressure is applied to produce an image. All methods in this experiment were performed in accordance with the relevant guidelines and regulations and conformed to the 3R principles and ARRIVE guidelines.

### 2.2 Dataset

In this study, we first created a pre-trained ultrasound dataset, consisting of five sub-datasets: AULI (Liver) (20), OCAU (Abdominal) (21), BUSI (Breast) (22), DDTI (Thyroid) (23), and EDCU (Heart, https://aimi.stanford.edu/datasets/echonet-dynamic-cardiac-ultrasound). The detailed information for these sub-datasets can be found in **Table 1**.

**Table 1:** The information of pretraining dataset.

Dataset name	Body part	Number of images	Number of classes	Name of classes	Number of instances per class	Width	Height
AULI	Liver	735	3	Benign/Malignant/Normal	200/435/100	874.33 ± 163.30	667.14 ± 107.78
OCAU	Abdominal	360	6	kidney/bladder/spleen/bowel/gallbladder/liver	60/60/60	64.00 ± 0.00	64.00 ± 0.00
BUSI	Breast	780	3	benign/malignant/normal	437/210/133	615.68 ± 121.98	501.45 ± 76.64
DDTI	Thyroid	301	2	malignant/normal	193/108	346.86 ± 36.30	278.55 ± 16.19
EDCU	Heart	218	2	closed/open	80/138	128.00 ± 0.00	128.00 ± 0.00

For our fine-tuning dataset—the lymph node ultrasound dataset—due to the small size of the dataset, we performed five-fold cross-validation. In each fold, the training set was used by the deep learning network to learn complex patterns that represent different phenotypes and disease changes, while the validation set was used to evaluate the model’s diagnostic and generalization performance. BLN represents benign lymph nodes (label 0), Lymphoma represents lymphoma (label 1), and MLN represents metastatic lymph nodes (label 2). 2D, elastography, and CDFI ultrasound images were input into the model and trained into three different sub-models.

All images were resized to a uniform size of 384x384 to ensure consistency in the input data. On the training set, we used random over-sampling to balance the data.

### 2.3 Algorithms

We selected the four most popular deep learning models: ConvNeXt, EfficientNet, ResNet, and ViT (24). Torchvision (https://pytorch.org/vision/stable/models.html) provides the performance of these mainstream models trained on the ImageNet-1K dataset. These four models perform excellently in image classification tasks and are widely applied in the field of medical image classification.

ConvNeXt is a modernized convolutional neural network, and in this study, we adopted the largest version of the ConvNeXt series (ConvNeXt-Large) from Torchvision. EfficientNet-B7 is the largest and most powerful version of the EfficientNet series, which utilizes a compound scaling strategy to jointly optimize network depth, width, and input resolution, greatly improving classification performance. Additionally, we introduced Vision Transformer (ViT), using 16×16 patch sizes to divide the images. ViT has gained widespread attention in recent years, effectively modeling global dependencies in images through the self-attention mechanism, exhibiting excellent performance. ResNet152 is a deeper variant in the ResNet series, employing deep residual learning to mitigate the vanishing gradient problem in deep networks. Although it has a higher computational complexity, it demonstrates strong performance in complex tasks by increasing the network depth.

For the pre-trained models, we replaced the original output head of ConvNeXt, EfficientNet, ViT, and ResNet with multi-layer perceptron (MLP) output heads for the pre-trained ultrasound dataset. When fine-tuning on the lymph node dataset, we retained the backbone weights of the previously trained models, removed the multi-task MLP head, and replaced it with a single MLP head consisting of two linear layers connected by ReLU layer. The first hidden layer contained 256 neurons, and the second output layer contained 3 neurons to meet the classification task requirements. This adjustment allowed the model to predict the nature of cervical lymph nodes.

### 2.4 Training and evaluation

In this study, we employed both “learning from scratch” and “pre-training and fine-tuning” strategies. Pre-training used multi-task pre-training, where we divided each of the five pre-trained ultrasound datasets into training and validation sets in a 4:1 ratio. For each epoch, we updated the weights by sequentially learning the five tasks, specifically using the cross-entropy loss function and the Adam optimizer with a learning rate of 0.00005 and a batch size of 16. The validation set was then used to evaluate early stopping, with a patience of 5 and the loss set to the average loss of the five tasks. Finally, the results from the best epoch of the validation set were used to evaluate the pre-training.

For fine-tuning, we applied five-fold cross-validation. For each fold, the same learning strategy was used as described above, but with only one MLP head for the output.

During evaluation, multiple performance metrics were employed, including AUC, accuracy, precision, recall, F1 score, and MCC, to compare the performance of pre-trained and non-pre-trained models. Additionally, confusion matrices and AUC curves were used to assess the classification accuracy across the four models and three imaging modalities.

### 2.5 Statistics analysis

R software (version 4.0.1) was used for statistical analysis. The relationships between categorical variables were assessed using the Chi-square test. For continuous variables, the Shapiro-Wilk test was first applied to check for normality. Data that followed a normal distribution were analyzed using the Independent t-test, while non-normally distributed data were analyzed using the Mann-Whitney U test. For comparisons across multiple groups, either One-way Analysis of Variance (ANOVA) or the Kruskal-Wallis H test was used, depending on the data distribution. A significance level of p < 0.05 or p < 0.01 was applied to all statistical tests.

### 2.6 Webserver implement

The backend of the CV4LymphNode website is powered by Django (https://www.djangoproject.com/), leveraging the model-view-controller (MVC) framework to provide real-time responsiveness. On the frontend, the site is built with React (https://react.dev/) and incorporates the Ant Design (Antd) UI library (https://ant.design/).

## 3 Results

### 3.1 Data source

**Figure 2:**
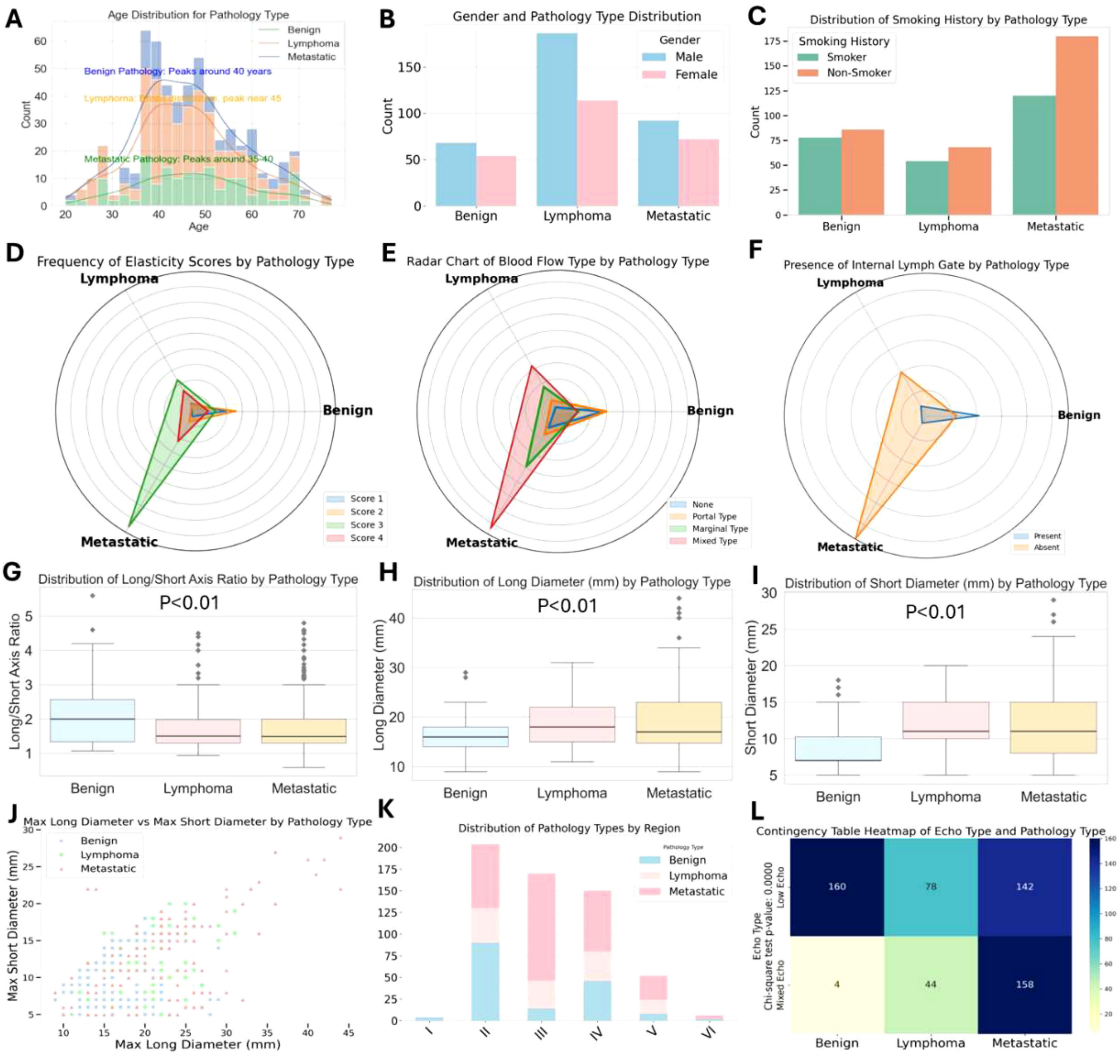
Distribution of 2D, CDFI and elastic imaging pathological feature across pathological types of cervical lymph nodes.

This study investigated the ultrasound characteristics of enlarged cervical lymph nodes and evaluated the potential of 2D ultrasound, color Doppler flow imaging (CDFI), and elastography in differentiating various pathological types of lymph nodes. A total of 586 patients were enrolled, comprising 300 cases of metastatic lymph nodes, 122 cases of lymphoma, and 164 cases of benign lymph nodes. Ultrasound examinations were conducted on all participants, and statistical analyses, including t-tests and chi-square tests, were performed to assess both clinical and ultrasound parameters. The results demonstrated significant differences in key ultrasound features, including elastography score, blood flow pattern, presence of hilum, and the long-to-short axis ratio, with all P-values < 0.01. In contrast, clinical characteristics such as age, gender, and smoking history showed no significant differences across the pathological groups (**Figures 2A–C**). These ultrasound parameters provide a reliable basis for distinguishing between different pathological types of lymph nodes and offer strong support for the development of machine learning-based classification models. Specifically, metastatic lymph nodes exhibited higher elastography scores, abnormal blood flow patterns, and a lack of hilum, while lymphoma and benign lymph nodes presented lower elastography scores and more uniform blood flow patterns. These findings suggest that ultrasound-based features can be effectively integrated into machine learning models for automated classification of cervical lymph node pathology, facilitating accurate diagnostic decision-making.

**Figures 2D-F** illustrate significant differences in elastography scores, blood flow types, and the presence of hilum among the different pathological groups (P-values < 0.01). Elastography scores reflect tissue stiffness, blood flow types indicate vascular patterns, and hilum presence or absence correlates with lymph node structure. Metastatic lymph nodes are most likely to exhibit elastography scores of 3 and 4, absence of hilum, mixed and peripheral blood flow patterns, while benign lymph nodes tend to show elastography scores of 1 and 2, presence of hilum, and blood flow patterns that are either absent or hilum-type. Lymphoma presents slightly weaker characteristics compared to metastatic lymph nodes.

Further analysis of the long-to-short axis ratio and the size parameters (long axis and short axis) revealed significant differences across the groups (P-values < 0.01), as shown in **Figures 2G-I**. Box plots demonstrated that metastatic lymph nodes had a significantly higher long-to-short axis ratio (mean 2.6) compared to benign lymph nodes (mean 1.2), reinforcing the importance of shape in pathological classification. Additionally, 68% of metastatic lymph nodes displayed mixed blood flow patterns, while lymphoma and benign lymph nodes typically exhibited more uniform or absent blood flow. These findings emphasize the critical role of ultrasound, particularly elastography and blood flow evaluation, in the differential diagnosis of cervical lymphadenopathy. The study provides a robust framework for distinguishing metastatic tumors, lymphoma, and benign conditions, offering valuable insights for the development of machine learning models aimed at automated disease classification (**Figures 2J, K, L**).

### 3.2 Pretrain results

**Figure 3:**
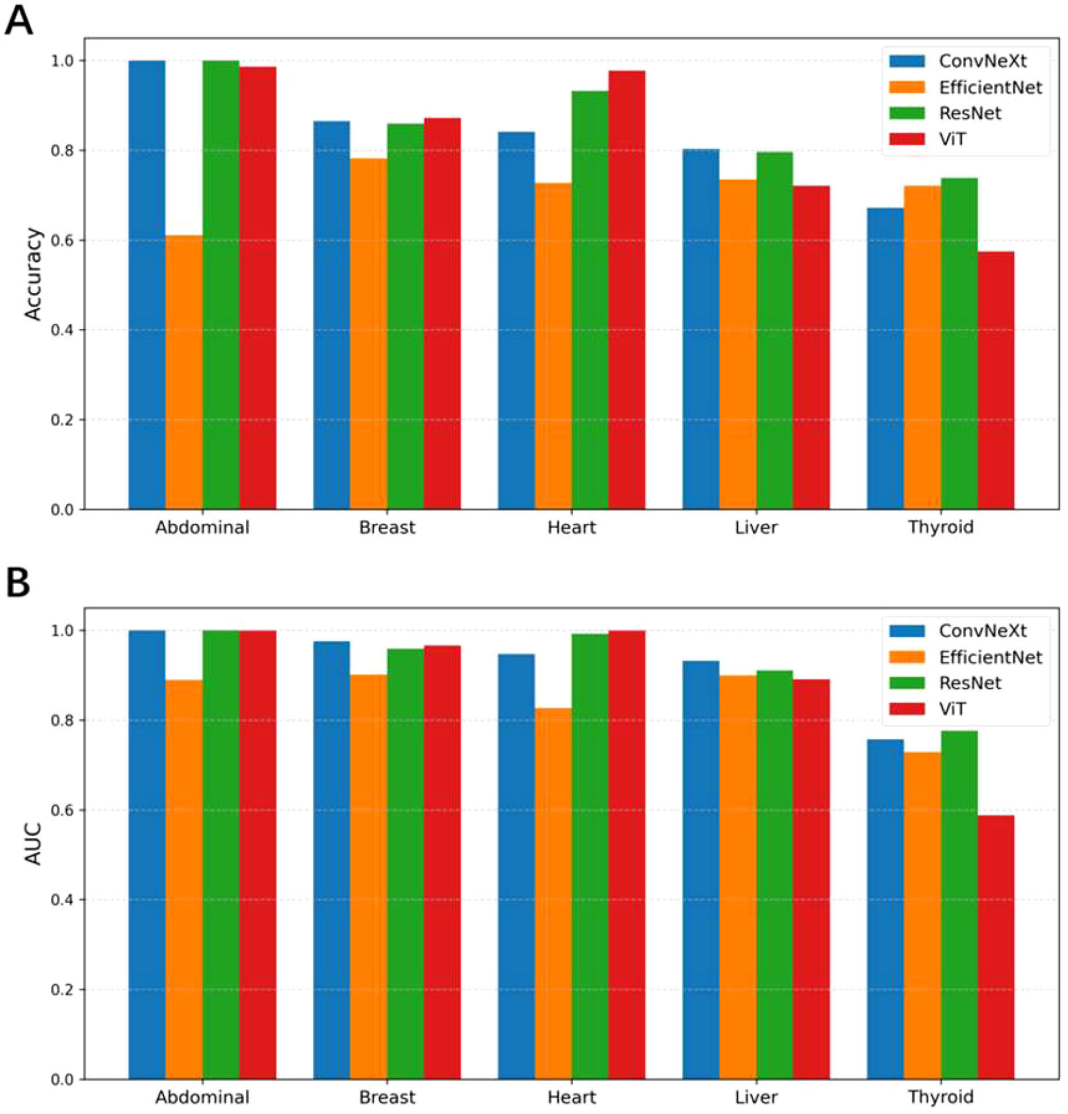
Comparison of classification accuracy **(A)** and AUC values **(B)** among different models (ConvNeXt, EfficientNet, ResNet, and ViT) across five pretrain ultrasound tasks (Abdominal, Breast, Heart, Liver, and Thyroid). Each group of bars represents the models’ performance on a specific task.

As shown in **Figure 3**, during the multi-task training on five ultrasound tasks, ConvNeXt and ResNet demonstrated strong performance, particularly achieving near-perfect results (AUC close to 1.000) in the Abdominal and Heart tasks. Among them, ConvNeXt performed exceptionally well in most tasks, especially in Abdominal, with perfect precision and recall. ResNet displayed more balanced performance across tasks, with particularly high AUC in Heart and Liver. In contrast, ViT performed well in most tasks but showed a significant decline in the Thyroid task, with an AUC of only 0.530, indicating that this task presents a challenge for the model. EfficientNet performed relatively poorly, especially in the Abdominal and Heart tasks, revealing limitations in these specific tasks. Overall, ConvNeXt and ResNet stood out in multi-task training, making them suitable for most ultrasound tasks, while ViT and EfficientNet may require optimization for specific tasks. Overall, our pre-training was successful, The specific confusion matrices and AUC curves can be found in **Supplementary Figure S1** and **Supplementary Figure S2**, while the tabular data is provided in **Supplementary Table S1**.

### 3.3 Finetune results

**Figure 4:**
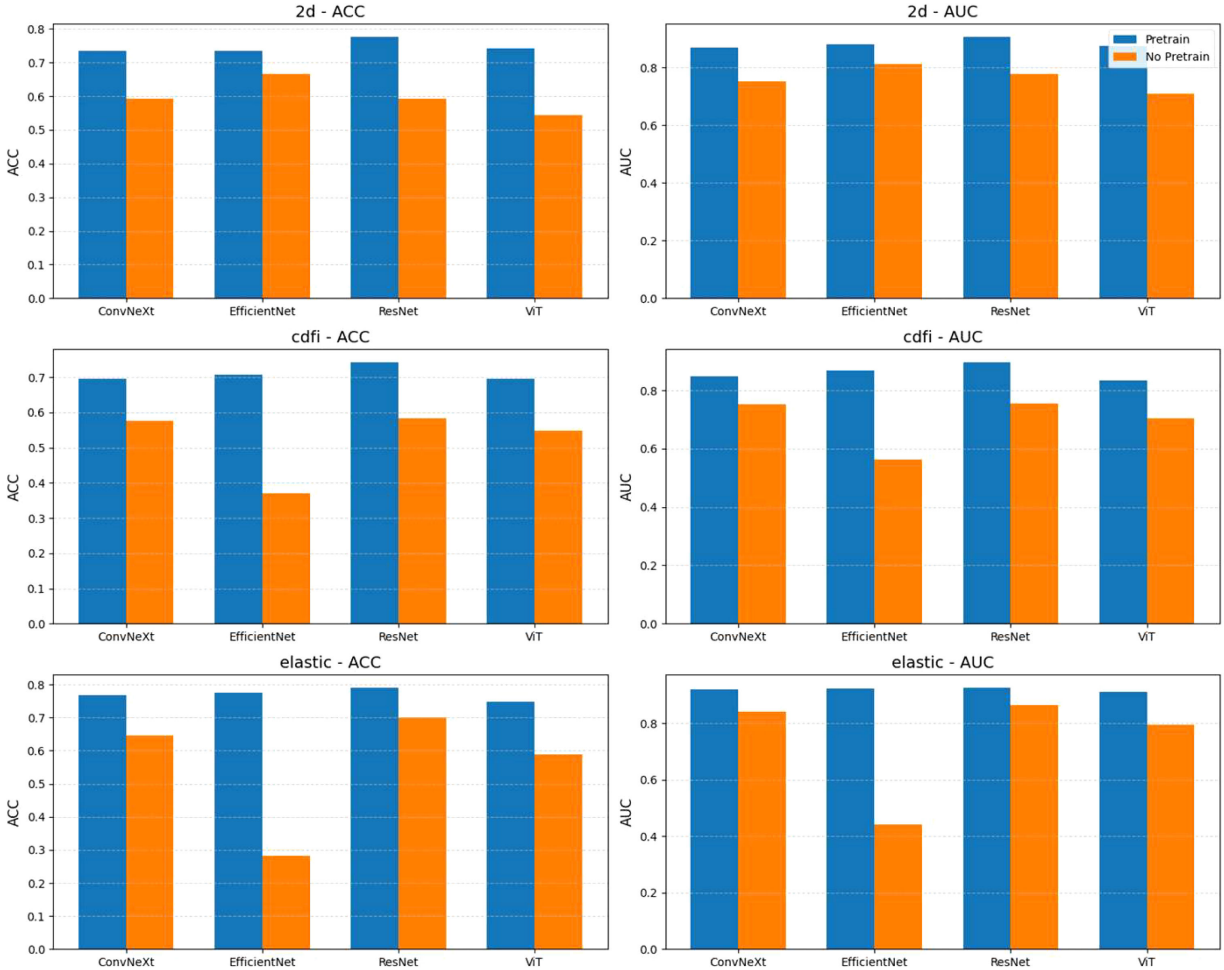
Comparison of model performance with and without pretraining across different tasks (2d, cdfi, elastic). Each subplot shows the accuracy (ACC) or area under the curve (AUC) achieved by four models (ConvNeXt, EfficientNet, ResNet, ViT) under two conditions: pretrained and not pretrained. Bars represent the mean metric values, with models grouped by task and evaluation metric. Pretraining generally improves performance across tasks and models.

As shown in **Figure 4**, from the experimental results with and without pretraining, pretraining significantly improved the performance of all models across different tasks. With pretraining, the ACC and AUC of each model showed notable improvements. For example, the average AUC of ConvNeXt, EfficientNet, ResNet, and ViT across the 2D, CDFI, and elastography tasks increased from 0.762, 0.694, and 0.736 without pretraining to 0.883, 0.862, and 0.920 with pretraining. Without pretraining, nearly all models had an ACC below 0.7 and an AUC below 0.8, while with pretraining, almost all models had an ACC above 0.7, and the AUC reached above 0.85. This indicates that pretraining on ultrasound images from other organs enhances the model’s generalization ability for downstream ultrasound image classification tasks.

Among all the tasks, elastography ultrasound images showed the best classification performance. Whether with or without pretraining, the overall ACC and AUC of the elastography task were higher than those of the 2D and CDFI tasks. For instance, the average AUC of the four models for elastography after pretraining was 0.92, higher than 0.883 for 2D and 0.862 for CDFI. However, we found that EfficientNet performed better in classifying 2D images from scratch, but performed poorly in CDFI and elastography tasks. However, pretraining significantly helped to improve its performance.

Among the four models, ResNet performed the best in all tasks. Whether with or without pretraining, ResNet showed stable and superior performance. In the 2D task, ResNet achieved an AUC of 0.906; in the CDFI task, it was 0.896; and in the elastography task, it was 0.925. Compared to the other models, ResNet achieved the highest AUC in all tasks, demonstrating its advantage in ultrasound image classification tasks.

**Figure 5:**
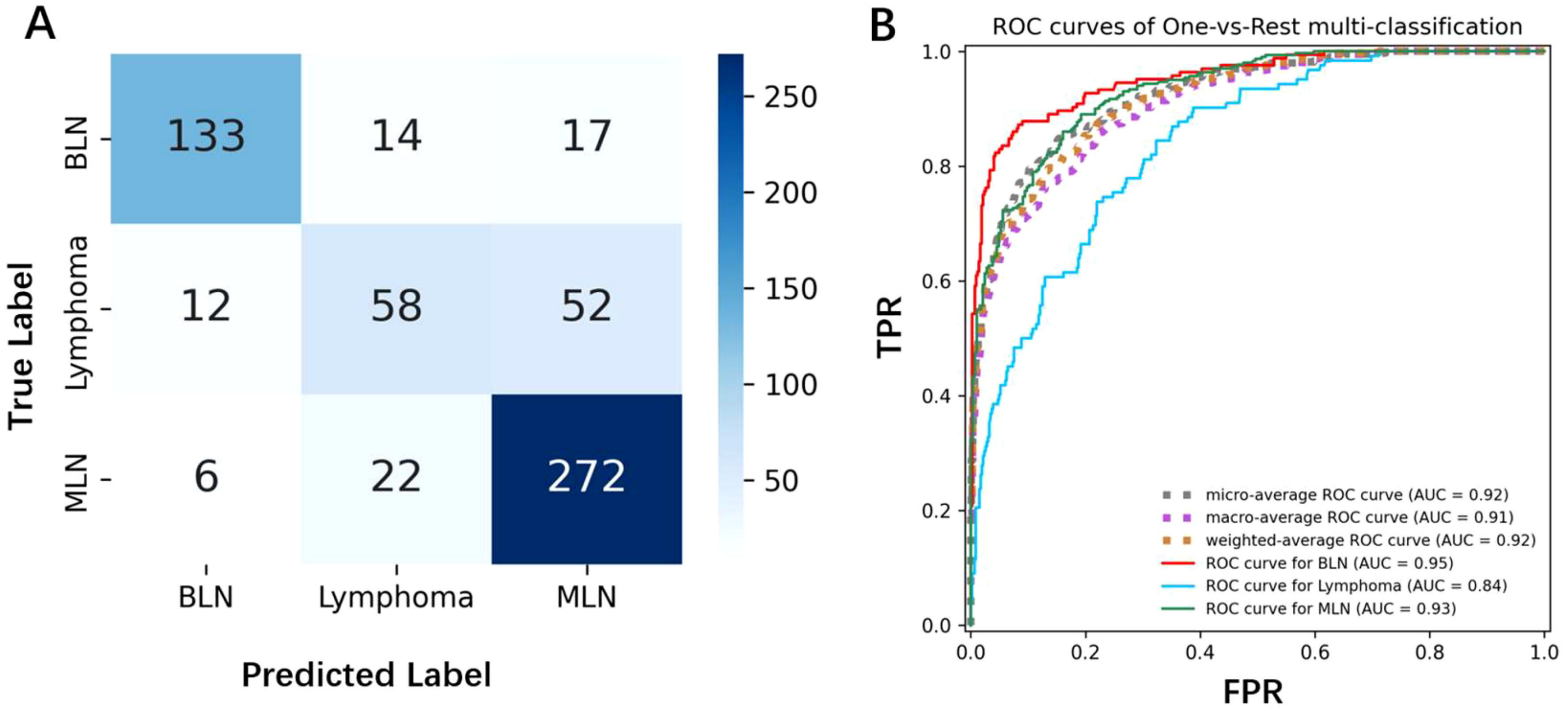
**(A)** The confusion matrix shows the classification results of the model for benign lymph nodes, lymphoma, and metastatic lymph nodes. The diagonal represents correctly classified samples, while the off-diagonal represents misclassified samples. **(B)** The AUC curve displays the model’s performance in the three classification tasks, with an AUC value closer to 1 indicating better model performance.

We selected the best-performing model—ResNet, pre-trained on five ultrasound datasets and fine-tuned on elastography images—as the candidate model. The confusion matrix and AUC curve for this model are shown in the **Figure 5**. The prediction of benign lymph nodes performed relatively well, with high precision and recall of 0.88 and 0.81, respectively. The precision and recall for lymphoma were lower, at 0.62 and 0.48, indicating that the model may confuse benign lymph nodes with lymphoma, particularly misclassifying lymphoma as metastatic lymph nodes. The recall for metastatic lymph nodes was relatively high at 0.91, but the precision was lower at 0.80, indicating that the model sometimes misclassifies other categories as metastatic lymph nodes. Lymphoma and metastatic lymph nodes can sometimes appear similar on imaging, as both can lead to lymph node enlargement and present as sclerosis, swelling, etc. Especially in the early stages, the distinction between the two may not be obvious, making it difficult for the model to differentiate. If the model has not learned the key distinguishing features between lymphoma and metastatic lymph nodes (such as irregular borders or density characteristics in lymphoma, or different shapes in metastatic lymph nodes), misclassification may occur. Misdiagnosing lymphoma as metastatic lymph nodes could lead to incorrect treatment plans. Lymphoma typically requires chemotherapy or radiotherapy, while metastatic lymph nodes are treated based on the primary tumor. Inappropriate treatment could delay recovery and negatively impact the patient’s health.

### 3.4 Webserver

**Figure 6:**
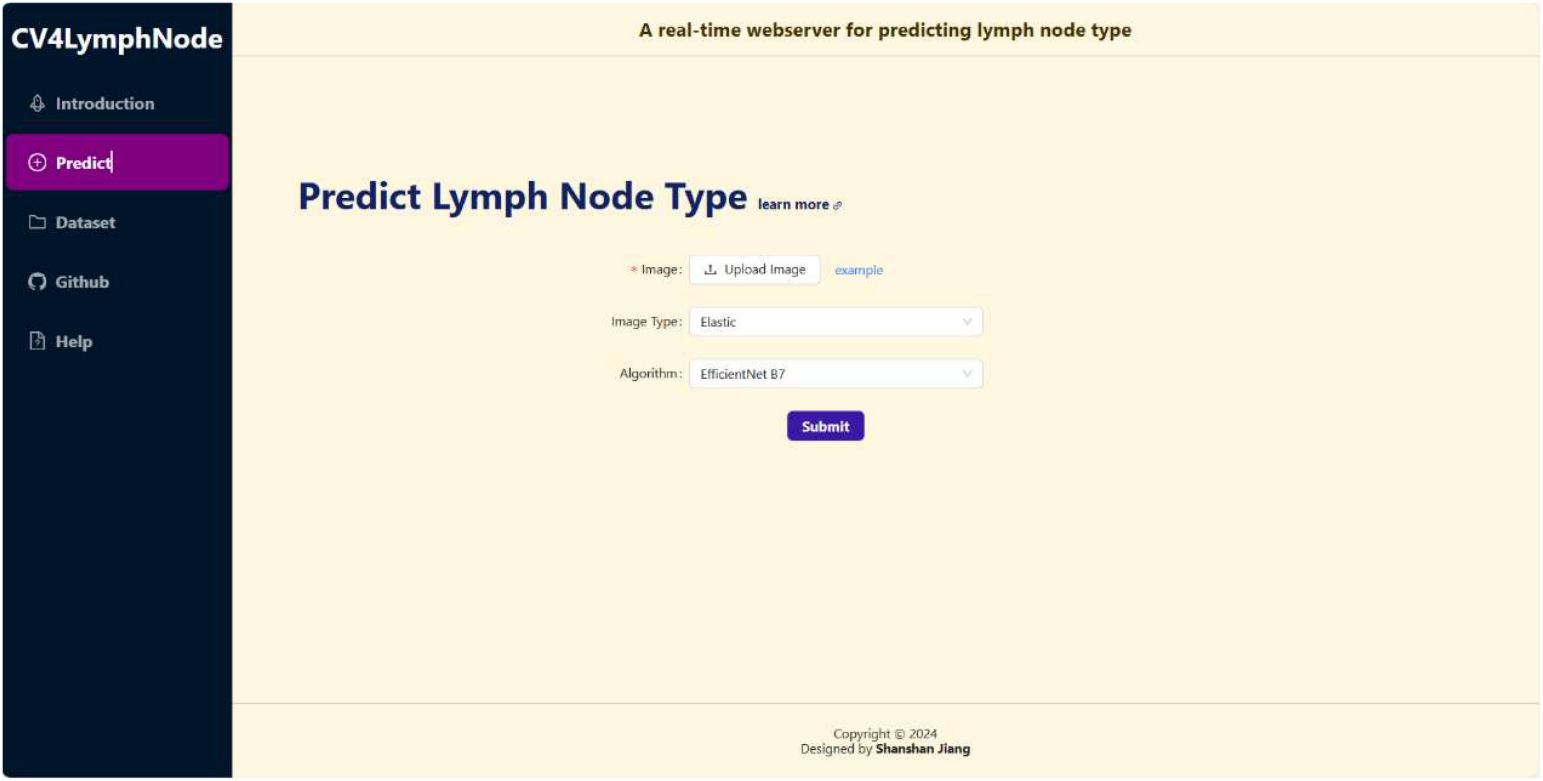
CV4LymphNode website interface.

We deployed the best performing ResNet model on the CV4LymphNode website, as shown in **Figure 6**. CV4LymphNode is a user-friendly website that allows users to upload 2D, elastic and CDFI lymph node ultrasound images and click Submit to get instant predictions. You can also navigate to data sets and code repositories.

### 3.5 Interpretability Analysis

**Figure 7:**
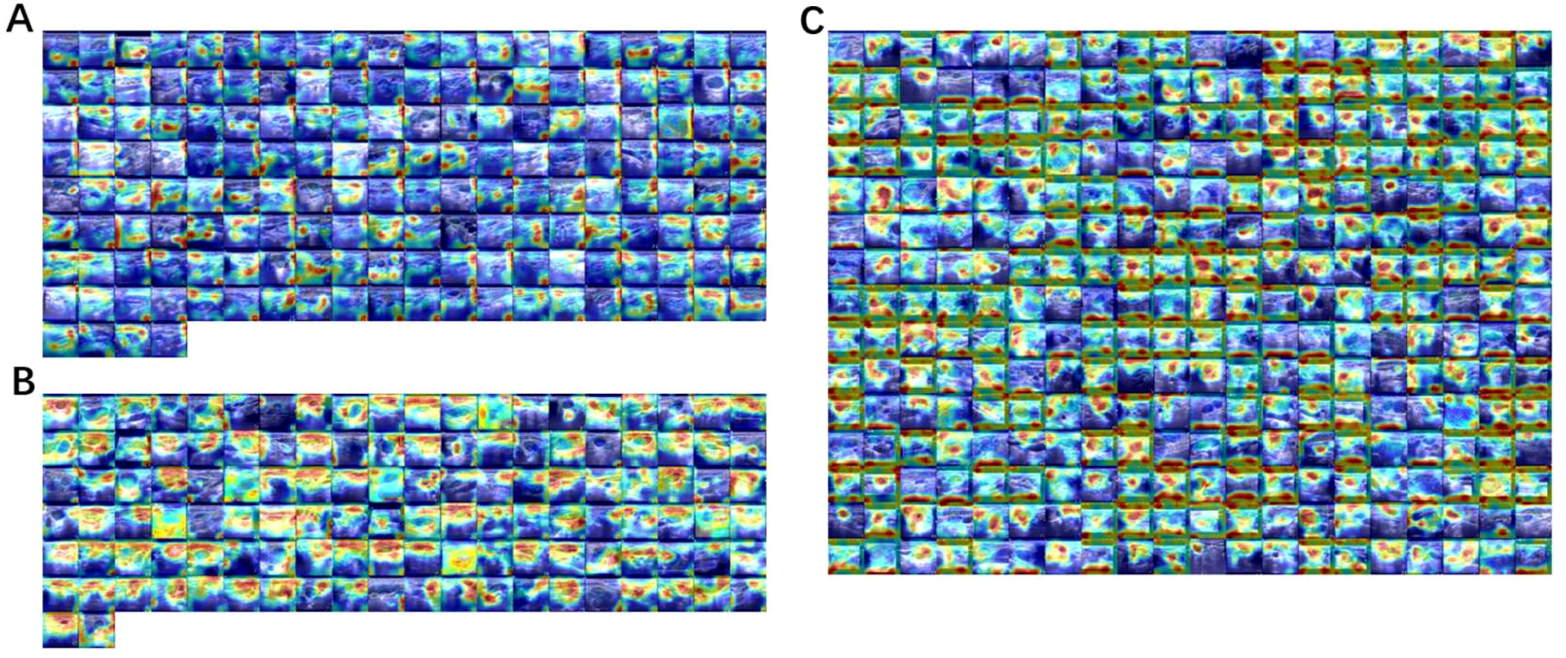
Using Grad-CAM to visualize the ResNet model on an elastic image dataset, **(A)** benign lymph nodes, **(B)** lymphoma, **(C)** metastatic lymph nodes. The redder the color, the more important the region, and the bluer the less important the region.

We used Grad-CAM (25) to analyze the visualizations of the ResNet model on the elastography image dataset to intuitively understand which areas of the image the model focuses on when making diagnostic decisions. This not only helps verify the model’s rationality and interpretability, but also provides valuable auxiliary information to clinicians, helping them better understand and trust the diagnostic results of the AI model. We found that the model focused on different features for the three different types of lymph nodes. For benign lymph nodes, the model focused on the non-lymph node areas of the image (**Figure 7A**). For lymphoma, ResNet concentrated on the lymphoma region, with little attention to other areas (**Figure 7B**). For metastatic lymph nodes, ResNet focused on the lymph node or surrounding textual information (**Figure 7C**). Through these phenotypes, we can understand how EfficientNet B7 identifies lymph node types, and this also provides us with certain references.

## 4 Discussion

In this retrospective study, we propose the use of deep learning-based Convolutional Neural Network (CNN) models to identify unexplained cervical lymphadenopathy through multimodal ultrasound (including 2D imaging, Color Doppler Flow Imaging (CDFI), and elastography). We conducted statistical analysis on the dataset used in this study, and the results are summarized as follows. Elastography score, tumor shape, and blood flow pattern were significantly associated with different tumor types (benign, lymphoma, and metastatic tumors). Variations in elastography scores reflect differences in tumor hardness, while the long-to-short axis ratio reveals the complexity of tumor shape, and differences in blood flow patterns highlight changes in blood demand and angiogenesis. The statistical analysis reached significant levels (p < 0.01). The CNN model achieved a diagnostic AUC of 0.925 in five-fold cross-validation. This model can effectively identify cervical lymph node lesions, supporting timely diagnosis and distinguishing different types of cervical lymphadenopathy. This method reduces puncture-related complications and provides valuable guidance for treatment plans and prognosis judgments.

In this study, we observed that benign lymph nodes are typically oval in shape with clear boundaries and exhibit typical lymphatic portal structures. In contrast, malignant lymph nodes often show significant changes in normal architecture, which can lead to alterations in morphology, internal echoes, and blood flow patterns. Ultrasound images show distinct features (24, 26). Malignant tumor cells release angiogenesis factors, leading to rich and chaotic blood flow in metastatic lymph nodes, primarily manifested as irregular, thickened, and twisted vessels in CDFI. Lymphoma also exhibits mixed features, mainly visible on CDFI. Therefore, significant differences in shape, boundaries, and CDFI are reflected in the results. Pathologically, lymphoma is characterized by tumor cell proliferation and infiltration, resulting in softer lymph nodes and lower elastography scores (27, 28). In contrast, metastatic lymph nodes are characterized by extensive vascular invasion, collagen generation, and calcification, leading to hardened texture and higher elastography scores. The unique ultrasound features and pathological characteristics of lymphoma contribute to the higher diagnostic accuracy of 2D imaging in distinguishing lymphoma compared to CDFI and elastography.

Deep learning models have several advantages in ultrasound image applications: (1) they can automatically learn useful features from ultrasound images, reducing the workload of manual feature engineering; (2) they reduce subjectivity and improve analysis accuracy; (3) they can handle images from different ultrasound devices, ensuring the model’s strong generalization capability; (4) they enable rapid diagnosis in seconds, significantly improving the efficiency of diagnosing complex diseases. In recent years, deep learning techniques have been increasingly applied to lymph node analysis. In the Introduction, we introduced three recent works on lymph node ultrasound classification, using ResNet, Swin Transformer, and CLA-HDM for lymph node ultrasound images. Due to the lack of publicly available datasets and algorithm codes, a fair comparison is difficult. However, compared to using ResNet alone, our ablation experiments show that pre-training improved the AUC of ResNet by 0.06. The Swin Transformer showed a 0.03 higher AUC than ResNet, suggesting that a pre-trained ResNet could improve the AUC by at least 0.6 compared to pure ResNet, and may perform at least as well as the Swin Transformer. Furthermore, compared to Swin Transformer, we conducted a more extensive evaluation of lymph node ultrasound images from CDFI and 2D modalities. In contrast to the binary classification of benign and malignant performed by CLA-HDM, we used a multiclass model to differentiate between benign, lymphoma, and metastatic lymph nodes, which better reflects the clinical diagnostic complexity. However, their use of dual-modality images with BUS and CDFI reminds us that using CDFI, 2D, and elastography images as inputs might also yield good results.

This study has some limitations. Ultrasound is a manual operation and inherently subjective, which may lead to differences in image quality between different doctors. For example, elastography, a technique used to assess tissue hardness, has broad applications in medicine but also has limitations, especially influenced by operator experience and device technical variability. First, the operator’s skill level directly impacts the scan quality, and different scanning methods and pressure applications may lead to inconsistent results. Second, different devices and imaging algorithms may result in differences in hardness measurement, and the maintenance and calibration of equipment may also affect the stability of results. Additionally, tissue heterogeneity and tumor type differences can affect the accuracy of elastography, especially in malignant tumors, where internal angiogenesis and fibrosis may lead to unstable measurements. Therefore, the diagnostic ability of elastography may vary in different patients and tumor pathological types, limiting its reliability for widespread application. Future research could consider incorporating other imaging technologies, such as microvascular imaging or contrast-enhanced ultrasound, or explore modality fusion for more comprehensive research approaches. Furthermore, since this study was conducted in three centers, collaboration with other centers for multi-center, large-sample prospective studies is recommended to further validate the applicability of these results in larger populations. Another limitation is the dataset’s imbalance. In this study, we used oversampling to balance the training set, and to prevent overfitting, we employed an early stopping strategy. Nevertheless, we acknowledge that our dataset is still biased and may not represent the typical three types of lymph nodes. Results also indicate that the distinction between metastatic and lymphoma lymph nodes needs improvement. In the future, we plan to expand the dataset to better represent the spatial distribution of lymph node ultrasound images.

In conclusion, our study confirms the feasibility of using deep learning CNN models based on ultrasound images to predict unexplained cervical lymphadenopathy. We provide metrics such as AUC and ACC for distinguishing between benign, lymphoma, and metastatic lymph nodes in multimodal ultrasound images. The results have significant clinical value in identifying these three diseases, and the diagnostic consistency meets clinical needs. Deep learning methods provide an objective and convenient predictive tool to assist doctors in making more accurate diagnoses.

## Data Availability

The datasets presented in this study can be found in online repositories. The names of the repository/repositories and accession number(s) can be found in the article/**Supplementary Material**.
