# Supplementary material for: Application of deep learning based on convolutional neural network model in multimodal ultrasound diagnosis of unexplained cervical lymph node enlargement
Source: Front Oncol. 2025 Jun 6;15:1542265. doi: 10.3389/fonc.2025.1542265 (PMC12178899; doi:10.3389/fonc.2025.1542265)
Supplement: Supplementary file 1 [file DataSheet1.docx]

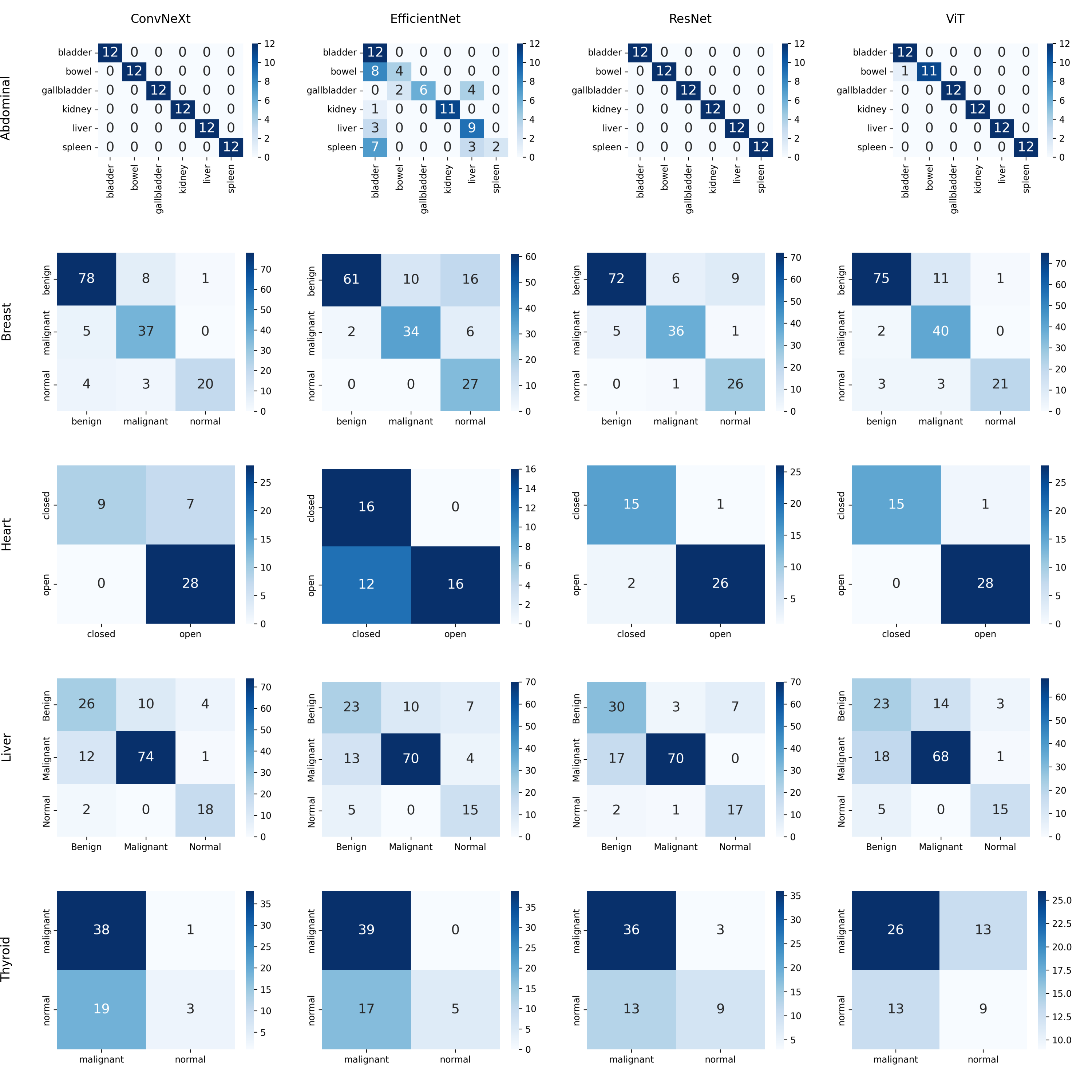


Figure S1. Confusion matrices of different models (ConvNeXt, EfficientNet, ResNet, and ViT) across five ultrasound tasks (Abdominal, Breast, Heart, Liver, and Thyroid). Each row represents a task, and each column represents a model, visualizing the classification performance.


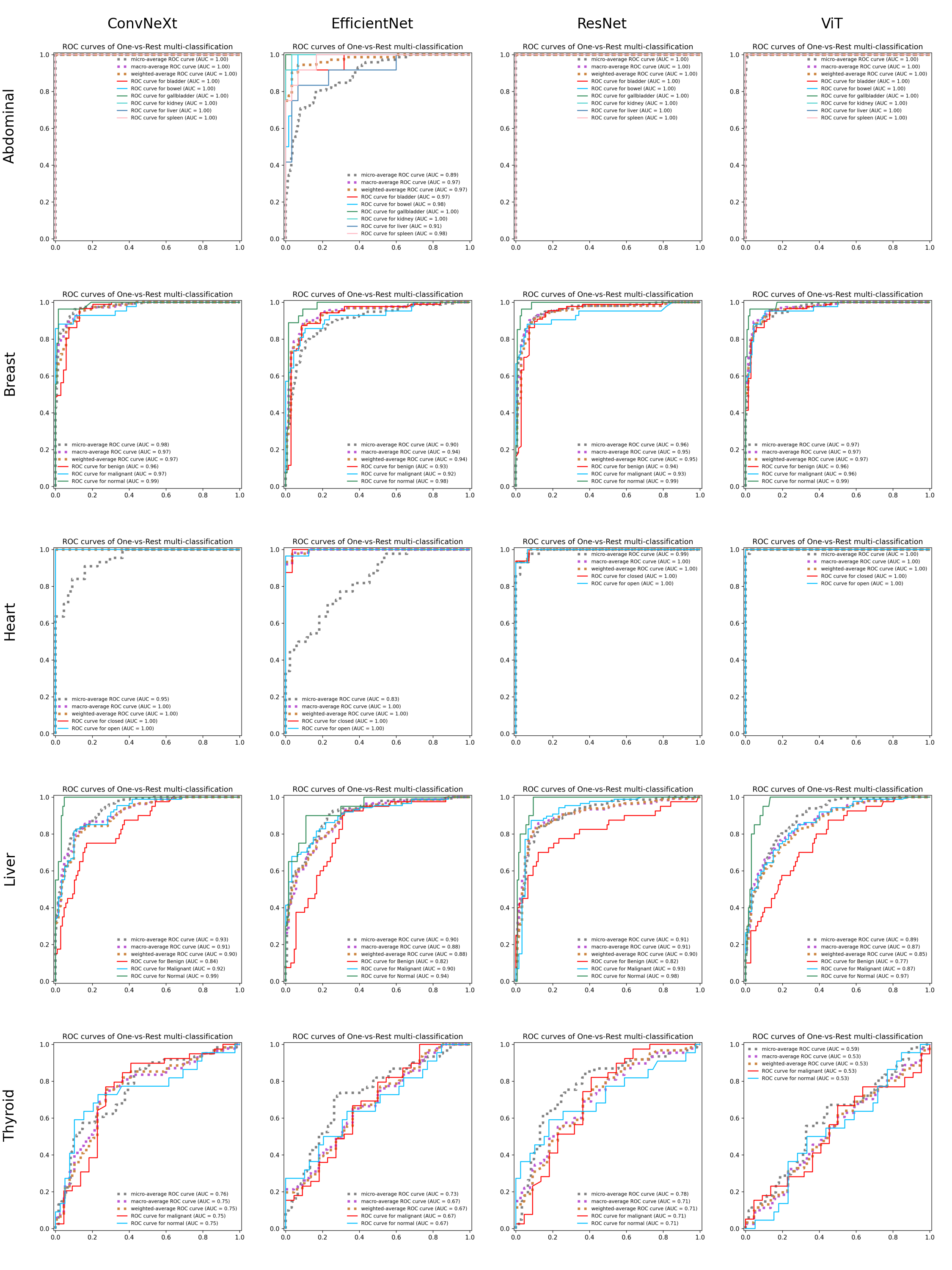


Figure S2. ROC curves of different models (ConvNeXt, EfficientNet, ResNet, and ViT) across five ultrasound tasks (Abdominal, Breast, Heart, Liver, and Thyroid). Each row represents a task, and each column represents a model, showing the classification performance for each class and overall AUC.


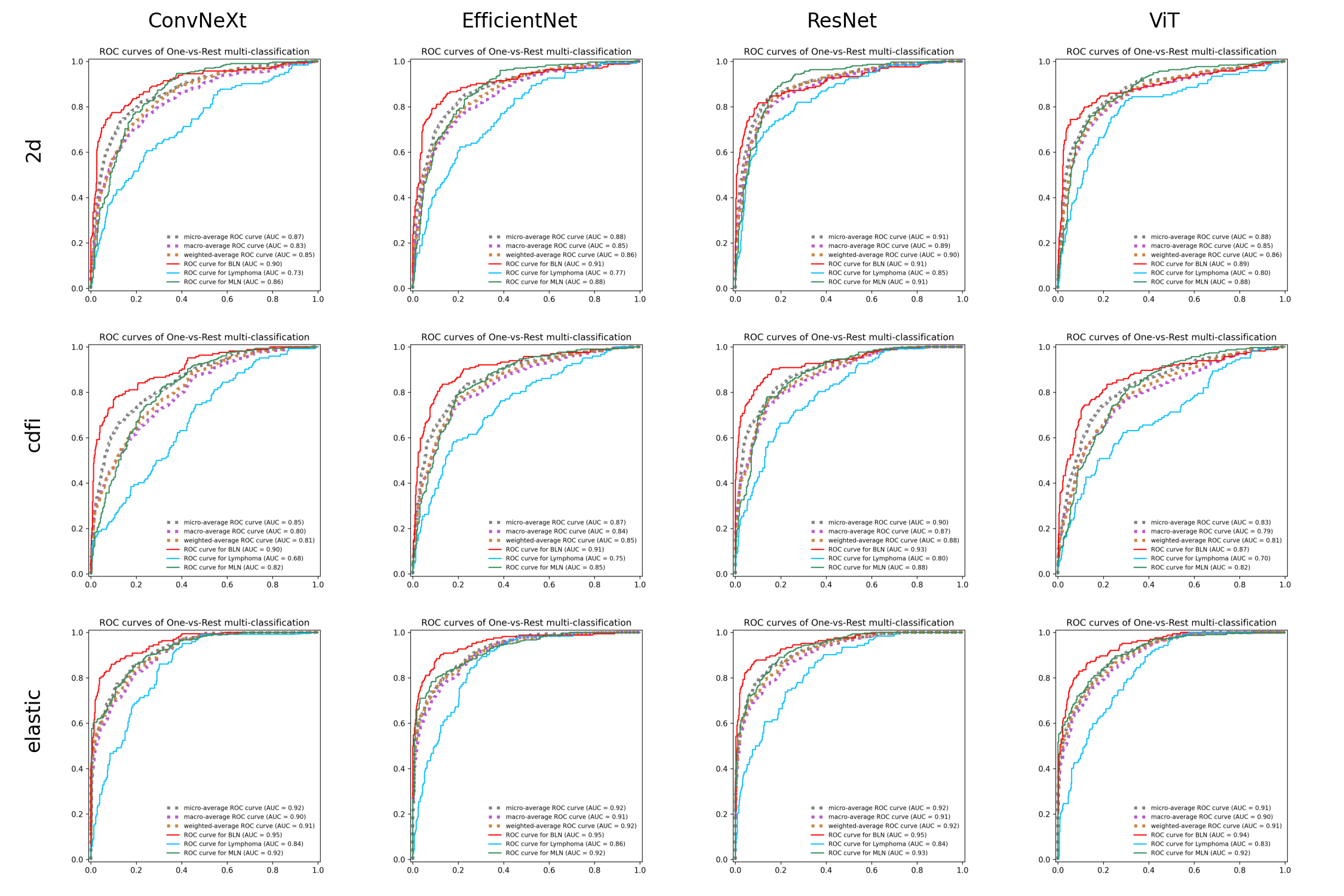


Figure S3. ROC curves of different models (ConvNeXt, EfficientNet, ResNet, and ViT) across three types of lymph node ultrasound datasets (2d, cdfi, and elastic). Each row represents a dataset type, and each column represents a model, showing the classification performance for each class and overall AUC.


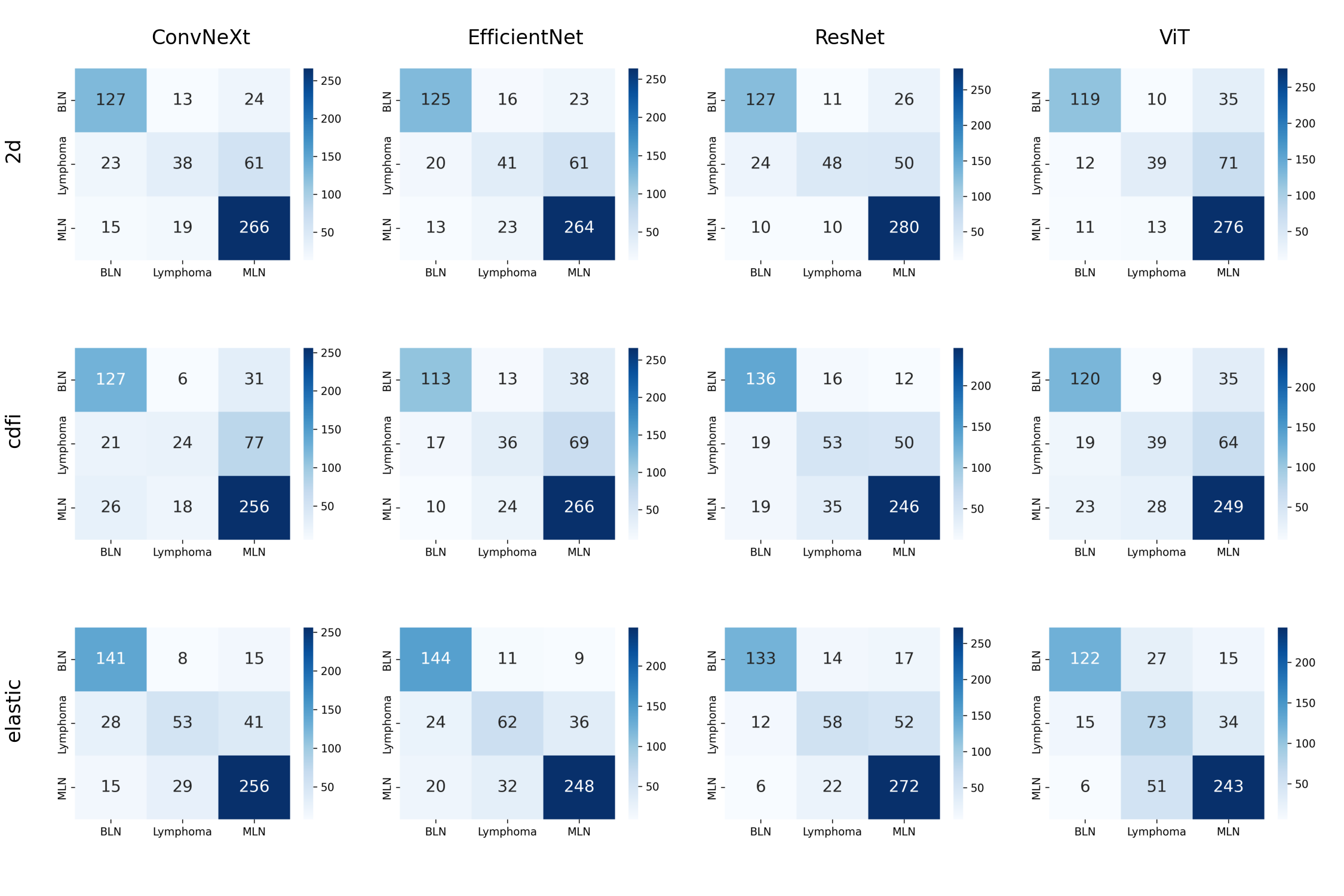


Figure S4. Confusion matrices of different models (ConvNeXt, EfficientNet, ResNet, and ViT) across three types of lymph node ultrasound datasets (2d, cdfi, and elastic). Each row represents a dataset type, and each column represents a model, visualizing the classification performance.


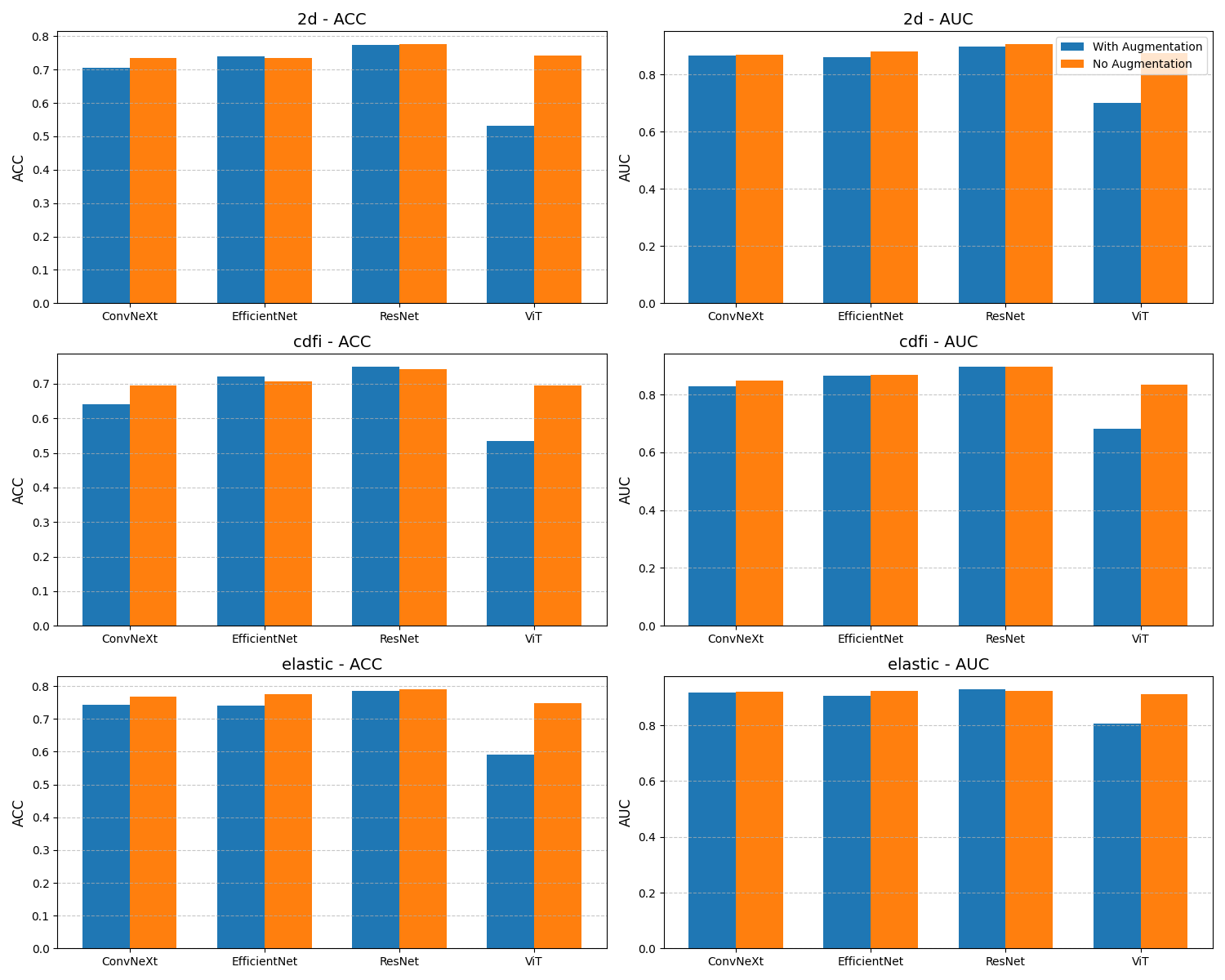


Figure S5. The impact of data augmentation versus no data augmentation on classification performance (ACC and AUC) for different models on various tasks. The chart displays the performance of different models (e.g., ConvNeXt, EfficientNet, ResNet, and ViT) in terms of AUC and ACC for each task (such as 2d, cdfi, and elastic). We applied eight image augmentation methods for data preprocessing: image cropping, random rotation, random vertical flip, random horizontal flip, random contrast variation, random brightness variation, random saturation variation, and random hue variation.

| Table S1. Performance comparison of four models (ConvNeXt, EfficientNet, ResNet, and ViT) across five ultrasound classification tasks in pretrain stage. | | | | | | |
| --- | --- | --- | --- | --- | --- | --- |
| Model | Task | PRE | REC | ACC | F1 | MCC |
| ConvNeXt | Abdominal | 1 | 1 | 1 | 1 | 1 |
| ConvNeXt | Breast | 0.872 | 0.865 | 0.865 | 0.866 | 0.771 |
| ConvNeXt | Heart | 0.873 | 0.841 | 0.841 | 0.827 | 0.671 |
| ConvNeXt | Liver | 0.805 | 0.803 | 0.803 | 0.803 | 0.652 |
| ConvNeXt | Thyroid | 0.697 | 0.672 | 0.672 | 0.589 | 0.215 |
| EfficientNet | Abdominal | 0.769 | 0.611 | 0.611 | 0.592 | 0.571 |
| EfficientNet | Breast | 0.843 | 0.782 | 0.782 | 0.789 | 0.686 |
| EfficientNet | Heart | 0.844 | 0.727 | 0.727 | 0.727 | 0.571 |
| EfficientNet | Liver | 0.749 | 0.735 | 0.735 | 0.739 | 0.543 |
| EfficientNet | Thyroid | 0.806 | 0.721 | 0.721 | 0.659 | 0.398 |
| ResNet | Abdominal | 1 | 1 | 1 | 1 | 1 |
| ResNet | Breast | 0.872 | 0.859 | 0.859 | 0.861 | 0.774 |
| ResNet | Heart | 0.934 | 0.932 | 0.932 | 0.932 | 0.856 |
| ResNet | Liver | 0.823 | 0.796 | 0.796 | 0.803 | 0.661 |
| ResNet | Thyroid | 0.74 | 0.738 | 0.738 | 0.714 | 0.401 |
| ViT | Abdominal | 0.987 | 0.986 | 0.986 | 0.986 | 0.984 |
| ViT | Breast | 0.887 | 0.872 | 0.872 | 0.874 | 0.791 |
| ViT | Heart | 0.978 | 0.977 | 0.977 | 0.977 | 0.951 |
| ViT | Liver | 0.734 | 0.721 | 0.721 | 0.726 | 0.51 |
| ViT | Thyroid | 0.574 | 0.574 | 0.574 | 0.574 | 0.076 |

| Table S2. Performance comparison of four models (ConvNeXt, EfficientNet, ResNet, and ViT) across 3 lymph node ultrasound classification tasks in finetune stage. | | | | | | | |
| --- | --- | --- | --- | --- | --- | --- | --- |
| Model | Task | PRE | REC | ACC | F1 | MCC | AUC |
| ConvNeXt | 2d | 0.735 | 0.735 | 0.735 | 0.735 | 0.56 | 0.87 |
| EfficientNet | 2d | 0.734 | 0.734 | 0.734 | 0.734 | 0.557 | 0.882 |
| ResNet | 2d | 0.776 | 0.776 | 0.776 | 0.776 | 0.63 | 0.906 |
| ViT | 2d | 0.741 | 0.741 | 0.741 | 0.741 | 0.568 | 0.875 |
| ConvNeXt | cdfi | 0.695 | 0.695 | 0.695 | 0.695 | 0.489 | 0.849 |
| EfficientNet | cdfi | 0.708 | 0.708 | 0.708 | 0.708 | 0.51 | 0.867 |
| ResNet | cdfi | 0.742 | 0.742 | 0.742 | 0.742 | 0.579 | 0.896 |
| ViT | cdfi | 0.696 | 0.696 | 0.696 | 0.696 | 0.493 | 0.835 |
| ConvNeXt | elastic | 0.768 | 0.768 | 0.768 | 0.768 | 0.62 | 0.921 |
| EfficientNet | elastic | 0.775 | 0.775 | 0.775 | 0.775 | 0.636 | 0.923 |
| ResNet | elastic | 0.79 | 0.79 | 0.79 | 0.79 | 0.653 | 0.925 |
| ViT | elastic | 0.747 | 0.747 | 0.747 | 0.747 | 0.596 | 0.911 |

| Table S3. Performance comparison of four models (ConvNeXt, EfficientNet, ResNet, and ViT) across 3 lymph node ultrasound classification tasks from scratch. | | | | | | | |
| --- | --- | --- | --- | --- | --- | --- | --- |
| Model | Task | PRE | REC | ACC | F1 | MCC | AUC |
| ConvNeXt | 2d | 0.594 | 0.594 | 0.594 | 0.594 | 0.297 | 0.752 |
| EfficientNet | 2d | 0.666 | 0.666 | 0.666 | 0.666 | 0.439 | 0.811 |
| ResNet | 2d | 0.594 | 0.594 | 0.594 | 0.594 | 0.31 | 0.778 |
| ViT | 2d | 0.543 | 0.543 | 0.543 | 0.543 | 0.201 | 0.708 |
| ConvNeXt | cdfi | 0.577 | 0.577 | 0.577 | 0.577 | 0.27 | 0.752 |
| EfficientNet | cdfi | 0.37 | 0.37 | 0.37 | 0.37 | 0.019 | 0.562 |
| ResNet | cdfi | 0.584 | 0.584 | 0.584 | 0.584 | 0.312 | 0.755 |
| ViT | cdfi | 0.549 | 0.549 | 0.549 | 0.549 | 0.183 | 0.705 |
| ConvNeXt | elastic | 0.645 | 0.645 | 0.645 | 0.645 | 0.438 | 0.841 |
| EfficientNet | elastic | 0.283 | 0.283 | 0.283 | 0.283 | 0.033 | 0.442 |
| ResNet | elastic | 0.7 | 0.7 | 0.7 | 0.7 | 0.505 | 0.865 |
| ViT | elastic | 0.589 | 0.589 | 0.589 | 0.589 | 0.339 | 0.795 |

| Table S4. Performance comparison of ResNet across elastic image dataset with 3 cropping methods in finetune stage. | | | | | | | |
| --- | --- | --- | --- | --- | --- | --- | --- |
| Model | Task | PRE | REC | ACC | F1 | MCC | AUC |
| ResNet | elastic | 0.79 | 0.79 | 0.79 | 0.79 | 0.653 | 0.925 |
| ResNet | elastic_middle | 0.746 | 0.746 | 0.746 | 0.746 | 0.577 | 0.891 |
| ResNet | elastic_small | 0.744 | 0.744 | 0.744 | 0.744 | 0.579 | 0.883 |

| **Table S5. Demographic and Clinical Characteristics of the Patients** | | | | | |
| --- | --- | --- | --- | --- | --- |
| **Pathology**  **Type** | **N** | **Male (%)** | **Female (%)** | **Age (Mean ± SD)** | **Major Pathological Subtypes** |
| Benign | 164 | 92 (56.1%) | 72 (43.9%) | 44.2 ± 9.4 | Reactive hyperplasia, Tuberculous，lymphadenitis, Kikuchi’s disease, Castleman’s disease, Granulomatous inflammation,Kimura disease, etc. |
| Lymphoma | 122 | 75 (61.5%) | 47 (38.5%) | 46.2 ± 9.6 | Diffuse large B-cell lymphoma, Mantle cell lymphoma, Follicular lymphoma, Hodgkin lymphoma, Peripheral T-cell lymphoma, etc. |
| Metastatic | 300 | 221 (73.7%) | 79 (26.3%) | 50.2 ± 9.9 | Lung cancer, Nasopharyngeal carcinoma, Breast cancer, Gastrointestinal cancers, Ovarian cancer, etc. |
